# Thrombophilia Testing in Stroke: A Case Report and Review of Evidence

**DOI:** 10.7759/cureus.50348

**Published:** 2023-12-11

**Authors:** Ranjit B Jasaraj, Ekaterina Proskuriakova, Suman Gaire, Aanchal Chaudhary, Pam Khosla

**Affiliations:** 1 Internal Medicine, Mount Sinai Hospital, Chicago, USA; 2 Internal Medicine, Marshfield Clinic Health System, Marshfield, USA; 3 Hematology and Oncology, Mount Sinai Hospital, Chicago, USA

**Keywords:** cryptogenic stroke, vertebral arterial dissection, thrombosis, testing for thrombophilia, stroke workup

## Abstract

Thrombophilia is commonly associated with venous thromboembolism, but its relationship with arterial thrombosis, specifically stroke, is not as clearly established. Several large studies have failed to establish a significant connection between inherited thrombophilia and stroke. While tests for Factor V Leiden mutation, prothrombin mutation, protein C deficiency, protein S deficiency, antithrombin deficiency, and antiphospholipid antibodies are typically done for thrombophilia diagnosis, there appears to be little or no correlation between these markers and stroke. In this article, we discuss a case of a 26-year-old male admitted with right neck pain that developed after playing basketball; he was found to have a right cerebellar infarction. He underwent extensive tests for hypercoagulable disorders, which were negative. We also review current evidence and reassess the value of thrombophilia testing in stroke patients.

## Introduction

Stroke is a major cause of morbidity and mortality in the United States. Every year, about 795,000 people develop strokes in the United States, of which around 610,000 are new strokes [1]. About 185,000 strokes, nearly one in every four, are in people who have already had a stroke [1]. Although the incidence of stroke rises with age, stroke can occur at any age. In 2014, 38% of individuals hospitalized for stroke were under the age of 65 [2]. Ischemic stroke accounts for 87% of all strokes [1], with cryptogenic stroke making up between 30-40% of those [3,4]. Cardiovascular risk factors play a significant role in the development of stroke. Although thrombophilia tests are frequently ordered for unexplained stroke, particularly in young adults, the evidence in support of testing for inherited thrombophilia in arterial thrombosis, especially in stroke, is minimal.

## Case presentation

A 26-year-old Hispanic male with a past medical history of migraine presented to the emergency department with acute onset of right-sided neck pain, dizziness, and weakness for one day. He developed dizziness and blurred vision while playing basketball. After resting for around 30 minutes, he drove back home. At home, he noticed that his gait was unstable, and he had weakness and numbness in his right upper and lower extremities. He was found confused by his family members and had multiple episodes of vomiting. He reported subjective weakness, dizziness, nausea, and headache.

Three weeks prior to this episode, he had visited the emergency department for right-sided neck pain and stiffness and was treated with muscle relaxants and naproxen. The patient did not have any personal or family history of connective tissue or hematological disorders. He did not have a prior history of head trauma, chiropractor visits, or illicit drug use.

Upon admission, the patient's vital signs were stable, and he was alert and oriented to person, place, and time. Cerebellar function tests were normal, and he did not present any focal neurologic deficits. His laboratory results were unremarkable.

**Figure 1:**
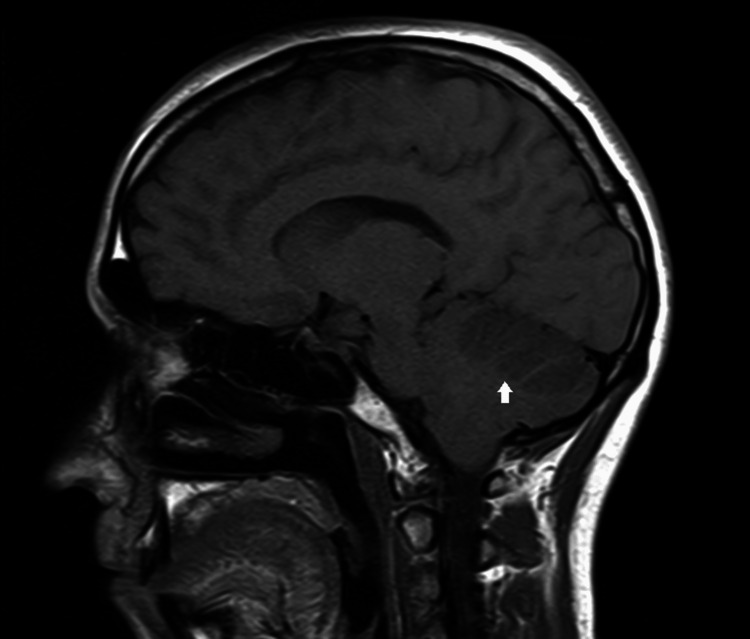
MRI brain (sagittal view, T1) showing right cerebellar ischemic stroke (white arrow)

**Figure 2:**
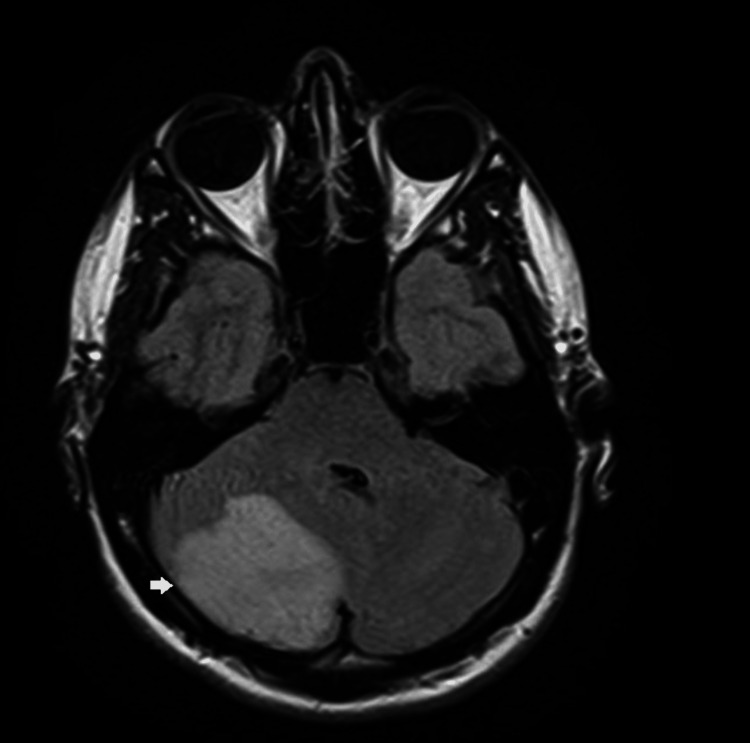
MRI brain (axial view) showing large right cerebellar ischemic stroke (white arrow)

CT head did not reveal any acute findings, and CT angiography (CTA) head and neck were initially reported as unremarkable. MRI was obtained, which showed large territory acute ischemia in the right cerebellar hemisphere and small territory acute ischemia in the left cerebellar hemisphere, and mass effect with sulcal effacement within the right cerebellar hemisphere and right-to-left shift at the tentorium cerebelli by 3 mm (Figures 1, 2). Transesophageal echocardiography did not reveal any shunt.

The patient was evaluated by various specialists, including neurology, neurosurgery, neuroradiology, and hematology. Several potential diagnoses were considered, such as vertebral artery dissection, mitochondrial encephalopathy, lactic acidosis and stroke-like syndrome, and thrombophilia. We sent extensive labs to detect any hypercoagulable disorder, including anticardiolipin antibody, lupus anticoagulant, anti-beta 2 glycoprotein antibody, factor V Leiden mutation analysis (FVL), homocysteine level, protein C (PC), protein S (PS), antithrombin activity (AT), and prothrombin G20210A, all of which were negative.

**Figure 3:**
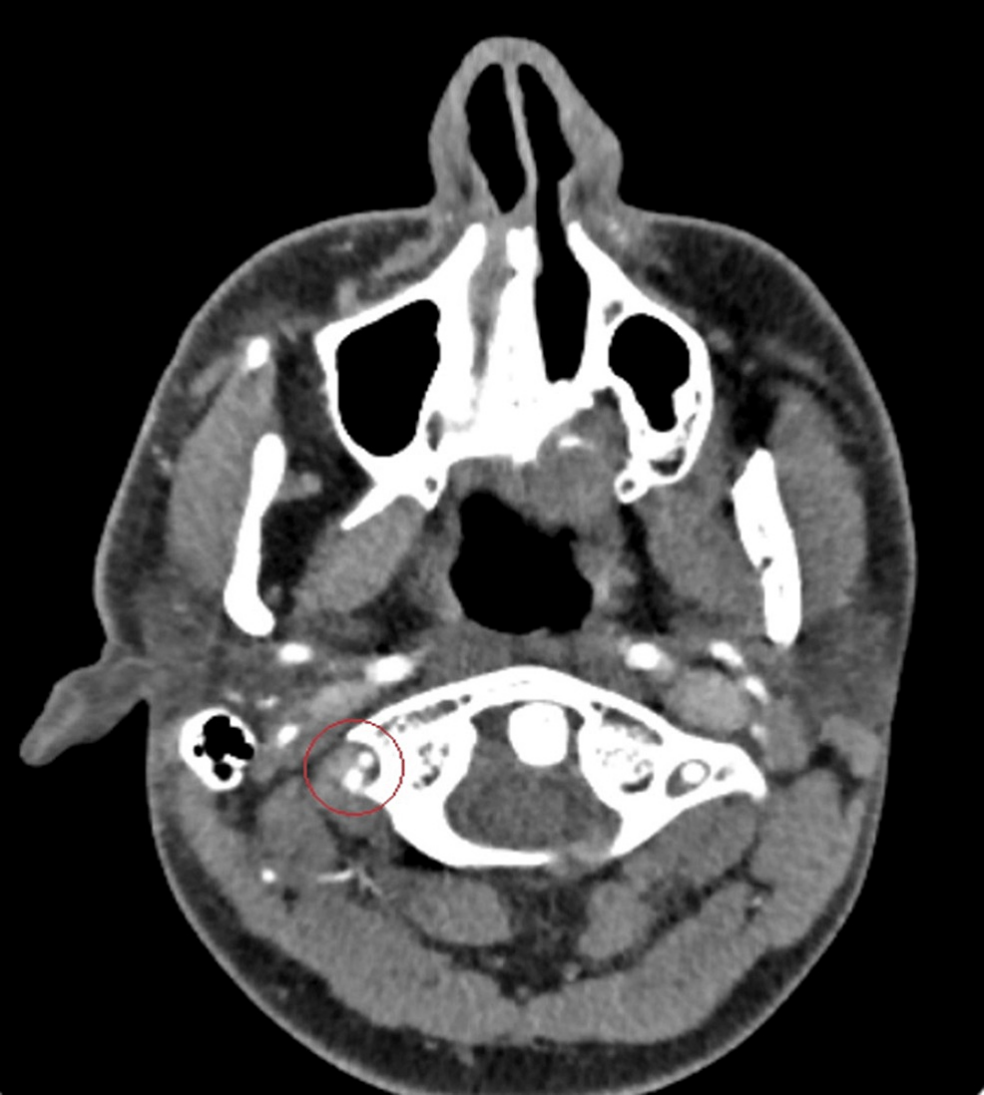
CTA head showing narrowing of the right vertebral artery (red circle) CTA: CT angiography

CTA neck and head were re-evaluated by neuroradiology, which revealed vertebral artery dissection (Figure 3). The patient was treated with aspirin and atorvastatin and managed with 3% hypertonic saline. He was not a candidate for neurosurgical intervention and was discharged on aspirin and atorvastatin. He was completely asymptomatic at discharge. The patient followed up with hematology service at four weeks and was found to be asymptomatic. Antiphospholipid labs were not repeated as initial labs were negative.

## Discussion

The role of thrombophilia in venous thromboembolism (VTE) is well-established, but its role in arterial thrombosis is not clear. While VTE is caused by a combination of blood flow stasis, hypercoagulability, and vessel wall damage, as suggested by Virchow's Triad, arterial thrombosis is mainly due to vessel wall abnormalities, particularly atherosclerosis. Multiple large studies have failed to show any significant association between stroke and inherited thrombophilia [5-7].

A panel of tests for FVL mutation, prothrombin G20210A mutation, PC deficiency, PS deficiency, AT deficiency, and antiphospholipid antibodies (APA) are usually ordered for thrombophilia workup. However, there is little or no association between inherited thrombophilia and stroke. A population-based case-control study involving 830 cases and 907 controls with first ischemic stroke at 15-49 years found no association between factor V Leiden and ischemic stroke [8]. Although a prior meta-analysis by the same author had concluded a positive association between factor V Leiden and ischemic stroke in young adults, many of the studies selected in the meta-analysis had cases with pre-existing suspected prothrombotic status or were limited to cryptogenic stroke. A significant association was not detected when the meta-analysis was limited to consecutive hospital admissions or neurology admissions [9]. The presence of FVL in atrial fibrillation (AFib) patients does not increase the risk of ischemic stroke [10]. The evidence to support an association between stroke and prothrombin G20210A mutation, PC deficiency, PS deficiency, or AT deficiency is limited. Therefore, inherited thrombophilia should not be tested for stroke [11,12].

For secondary stroke prevention, the American Heart Association guideline for stroke prevention recommends antiplatelet therapy in patients with inherited thrombophilia if there is no previous history of thrombosis and the source of stroke is unknown despite diagnostic tests [13]. It is important to note that detecting thrombophilia at the time of the acute event does not change management. If testing is considered due to a high degree of suspicion for thrombophilia, tests for PC, PS, or AT should be deferred or repeated at least four to six weeks after acute stroke as these protein levels may be altered during the acute phase of the stroke. However, follow-up of abnormal results is generally poor, which limits the impact of the thrombophilia testing policy [14].

Testing for thrombophilia markers like PC, PS, AT, and APA soon after a thrombotic event would increase the likelihood of false-positive results [15]. When considering testing for these markers after a thrombotic event, it is important to carefully weigh the need for the test and how to interpret positive and negative results. Testing too soon after the event could lead to false-positive results, causing uncertainty about the test's validity and the need for repeat tests and increased costs. Additionally, testing for factor V Leiden and the prothrombin gene may not accurately establish thrombophilia as the cause of stroke, as other factors could be contributing to the stroke.

Recent studies have shed light on the prevalence and clinical significance of thrombophilia markers in stroke patients. A study by Hankey et al. reported that one in seven patients tested positive for thrombophilic disorder after first acute ischemic stroke [7]. In another study involving 1900 patients admitted for acute ischemic stroke, 10% were tested for thrombophilia, of which 72% had at least one abnormal result and only 2% of tested patients had a change in management after testing positive for thrombophilic disorder [16]. In a retrospective study involving stroke patients with age <60 years, 14% tested positive for thrombophilia out of the 57% who were tested. Of these, 8% were positive for APA, 4% were homozygous or heterozygous for FVL or PGM, and 3% were protein C, S, or AT deficient [14].

Antiphospholipid syndrome (APS) has been identified as a significant risk factor for stroke, particularly in young adults. A systematic review of stroke in young adults aged less than 50 years showed that APA were present in 17.2% of stroke cases and increased the risk of cerebrovascular events by 5.48 times [17]. Another systematic review reported the frequency of APA to be 13.5% in stroke patients [18]. The risk of stroke in these patients depends on the positivity of the APA. Isolated lupus anticoagulant. and triple positivity are associated with the highest risk of stroke [19]. Hence, testing for APS in young patients with stroke without traditional stroke risk factors and no clear source of stroke is reasonable. A 2021 guideline by the American Hospital Association (AHA) recommends the use of antiplatelet therapy alone to reduce the risk of recurrent stroke in patients with isolated APA and anticoagulation with warfarin if the criteria for APS are fulfilled [13]. It is important to note that APA can be transiently positive after a stroke and should be tested at least 12 weeks later to establish the diagnosis of APS per Sapporo criteria. Therefore, careful consideration and interpretation of thrombophilia markers and APS testing are crucial in the management of stroke patients.

## Conclusions

Thrombophilia testing for inherited thrombophilia markers is not recommended in routine stroke evaluation, as the evidence for their association with stroke is minimal. However, in the case of young stroke patients who do not exhibit traditional risk factors and do not have a clear source of stroke, testing for APA may be appropriate if there is a concern for APS.

## References

[1] Tsao CW, Aday AW, Almarzooq ZI (2022). Heart disease and stroke statistics-2022 update: a report from the American Heart Association. Circulation.

[2] Jackson G, Chari K (2019). National Hospital Care survey demonstration projects: stroke inpatient hospitalizations. Natl Health Stat Report.

[3] Yaghi S, Bernstein RA, Passman R, Okin PM, Furie KL (2017). Cryptogenic stroke: research and practice. Circ Res.

[4] Li L, Yiin GS, Geraghty OC, Schulz UG, Kuker W, Mehta Z, Rothwell PM (2015). Incidence, outcome, risk factors, and long-term prognosis of cryptogenic transient ischaemic attack and ischaemic stroke: a population-based study. Lancet Neurol.

[5] Cushman M, Rosendaal FR, Psaty BM (1998). Factor V Leiden is not a risk factor for arterial vascular disease in the elderly: results from the Cardiovascular Health Study. Thromb Haemost.

[6] Ridker PM, Hennekens CH, Lindpaintner K, Stampfer MJ, Eisenberg PR, Miletich JP (1995). Mutation in the gene coding for coagulation factor V and the risk of myocardial infarction, stroke, and venous thrombosis in apparently healthy men. N Engl J Med.

[7] Hankey GJ, Eikelboom JW, van Bockxmeer FM, Lofthouse E, Staples N, Baker RI (2001). Inherited thrombophilia in ischemic stroke and its pathogenic subtypes. Stroke.

[8] Hamedani AG, Cole JW, Cheng Y (2013). Factor V leiden and ischemic stroke risk: the Genetics of Early Onset Stroke (GEOS) study. J Stroke Cerebrovasc Dis.

[9] Hamedani AG, Cole JW, Mitchell BD, Kittner SJ (2010). Meta-analysis of factor V Leiden and ischemic stroke in young adults: the importance of case ascertainment. Stroke.

[10] Hald EM, Løchen ML, Mathiesen EB, Njølstad I, Brækkan SK, Hansen JB (2020). Factor V leiden is associated with atrial fibrillation, but not ischemic stroke in atrial fibrillation. The Tromsø Study. Res Pract Thromb Haemost.

[11] Boekholdt SM, Kramer MH (2007). Arterial thrombosis and the role of thrombophilia. Semin Thromb Hemost.

[12] Carroll BJ, Piazza G (2018). Hypercoagulable states in arterial and venous thrombosis: when, how, and who to test?. Vasc Med.

[13] Kleindorfer DO, Towfighi A, Chaturvedi S (2021). 2021 guideline for the prevention of stroke in patients with stroke and transient ischemic attack: a guideline from the American Heart Association/American Stroke Association. Stroke.

[14] Alakbarzade V, Taylor A, Scully M, Simister R, Chandratheva A (2018). Utility of current thrombophilia screening in young patients with stroke and TIA. Stroke Vasc Neurol.

[15] Favaloro EJ, McDonald D, Lippi G (2009). Laboratory investigation of thrombophilia: the good, the bad, and the ugly. Semin Thromb Hemost.

[16] May J, Lin C, Martin K, Taylor LJ, Gangaraju R (2019). Thrombophilia testing in hospitalized patients with acute ischemic stroke: an opportunity for hematology input. Blood.

[17] Sciascia S, Sanna G, Khamashta MA, Cuadrado MJ, Erkan D, Andreoli L, Bertolaccini ML (2015). The estimated frequency of antiphospholipid antibodies in young adults with cerebrovascular events: a systematic review. Ann Rheum Dis.

[18] Andreoli L, Chighizola CB, Banzato A, Pons-Estel GJ, Ramire de Jesus G, Erkan D (2013). Estimated frequency of antiphospholipid antibodies in patients with pregnancy morbidity, stroke, myocardial infarction, and deep vein thrombosis: a critical review of the literature. Arthritis Care Res (Hoboken).

[19] Ruiz-Irastorza G, Cuadrado MJ, Ruiz-Arruza I (2011). Evidence-based recommendations for the prevention and long-term management of thrombosis in antiphospholipid antibody-positive patients: report of a task force at the 13th International Congress on Antiphospholipid Antibodies. Lupus.

